# Supplementary material for: Human-environment feedback and the consistency of proenvironmental behavior
Source: PLoS Comput Biol. 2023 Sep 18;19(9):e1011429. doi: 10.1371/journal.pcbi.1011429 (PMC10538744; doi:10.1371/journal.pcbi.1011429)
Supplement: Attachment — Submitted filename: Answer_reviewers_Article PLOS .pdf [file pcbi.1011429.s004.pdf]

Dr. Christian Hilbe
Academic Editor, *PLOS Computational Biology*

Dr. James O'Dwyer
Section Editor, *PLOS Computational Biology*

Ref.: Manuscript PCOMPBIOL-D-22-01352

March 21, 2023

Dear Dr. Hilbe and Dr. O'Dwyer,

We thank you for gathering insightful and constructive reviews of our manuscript "Human-environment feedback and the consistency of proenvironmental behavior" and for giving us the opportunity to submit a revised version. In our revision, we addressed all points raised by the three Reviewers, as well as your editorial concerns.

As you pointed out in your decision letter, Reviewer #1 raised relevant questions about some of the model assumptions and called for better communicating the originality and innovation of the work. To achieve this, we made several substantial changes. First and foremost, we clarified our definition of 'behavior consistency' by providing a mathematical characterization based on the analysis of the model's stochastic fluctuations. This highlights the originality of our mathematical approach, which starts with a microscopic, individual-based stochastic model, and establishes a macroscopic approximation comprising ordinary differential equations for the deterministic part of the dynamics, and a Ornstein-Uhlenbeck type process for the stochastic fluctuations. Using this rigorous characterization of behavior consistency, we clarified and streamlined the presentation of the results, updated figure 3 to provide a more comprehensive picture of the effect of key parameters on the fluctuations, and added the new figure 5 to illustrate the effect of fluctuations on the system dynamics in the bistable case.

We also explained the originality of our model with respect to other behavior-environment models that pertain to evolutionary games. In such eco-evolutionary games, the environmental feedback is mediated by the game payoffs, and its effect is thus tied to encounters among individuals. In contrast, our model assumes two channels for feedbacks: the social context feeds back via social encounters, while the environmental context feeds back via the individuals' own assessment of the environmental state. In this work, our primary motivation was to disentangle the effect of these feedbacks (positive and negative, respectively) on the consistent adoption of costly proenvironmentalism. We now note that this question and our treatment align with the conceptual framework, rooted in behavioral economics and cognitive psychology, that Caroline Schill and collaborators (2019 *Nature Sustainability*) put forth recently to provide "a more dynamic understanding of human behaviour for the Anthropocene".

These responses to the Reviewer 1's comments also address the main concerns of Reviewer 3, who called for clarifying the definition of behavior consistency and the main conclusions

of the study. Regarding the latter, we thoroughly revised the Abstract and strived to provide a new, crisper concluding paragraph. Please see our point-by-point response for more detail.

In our revised manuscript all changes appear in red. We updated the figures (including the new figure 5) and the supplementary material. Also, the code used to derive the results has been uploaded to PLOS platform with a readme file that explains how to use it.

We hope that our response and revised manuscript meet your expectations and we look forward to your decision.

Sincerely,

Claire Ecotière (corresponding author)
Sylvain Billiard
Jean-Baptiste André
Pierre Collet
Régis Ferrière
Sylvie Méléard

Point-by-point response

We appreciate your time and effort to provide us with insightful and constructive comments. We addressed all of them as critically and thoroughly as we could. This led us to make substantial changes to the manuscript. We feel that the paper is much improved as a result, in terms of clarity and potential impact. Thank you for your suggestions and guidance.

Hereafter your comments are in black; our responses appear in blue, and quotes from the revised manuscript are in purple.

Reviewer #1

Summary

In this manuscript, the authors engage in the question of how pro-environmental behavioral consistency is affected by social norms and the individual assessment of the environmental state and how, in turn, behavior affects the perceived environmental state. To answer this question, they develop an individual-based model which captures behavior change and environmental perception via transition rates or transition timescales. The majority of the analysis is performed via a set of deterministic differential equations, which approximate the individual-based stochastic model and resemble imitation/replicator dynamics from evolutionary game theory. Behavioral consistency is operationalized by the population equilibrating to either fully adopting option B (baseline business-as-usual impact) or option A (active pro-environmentalism). The authors show that the interaction of social norms and individual environmental assessment is more important for behavioral consistency than the timescale of how the perception of the environment changes.

We appreciate your summary of our work. Regarding the main results, we would add that the interaction of social influence and individual environmental assessment makes the consistent adoption of proenvironmental behavior possible when neither one, taken in isolation, would. This point is now made more clearly in the Abstract (lines 13-15) and Concluding remarks (lines 433-436).

Overall assessment

I appreciate how the authors motivate their model, coming from a relevant question in the sustainability discourse. The manuscript is clearly written. The model is derived and presented with sufficient mathematical detail and analyzed intensively. However, I believe, quite some improvements can be made. More work is needed to communicate the originality and innovation of this work (Comments 1-2), elaborate on the model design and analysis (Comments 3-4), and discuss how the model links to reality (Comments 5-6).

We are grateful for all your suggestions, which address central aspects of our analysis and presentation.

Comments

1. I still struggle to see what the main contribution of this work is. Is it a methodological advancement for human-environment modeling? Or a contribution to the question of why people behave inconsistently in their pro-environmental behavior? I believe the authors need to elaborate more on this point and critically discuss the main contribution of their work.

Understanding the conditions under which proenvironmental behaviors can be adopted consistently is the primary motivation of our work. Although our model is minimal, the analysis yields novel insights. The model also provides a framework for future extensions and a mathematical methodology, by deriving macroscopic dynamical equations (behavior frequency and their stochastic fluctuations) from a microscopic description of individuals (agents) interacting among themselves and with their environment.

To clarify this, we made several modifications and additions throughout the manuscript.

- In the Abstract, the central question is now stated more clearly (lines 3-7):

Here, we develop a mathematical model of a simple behavior-environment feedback loop to ask how the individual assessment of the environmental state combines with social interactions to influence the consistent adoption of proenvironmental behavior, and how this feeds back to the perceived environmental state.

- The Introduction ends with a clear statement of the general goal of the study and specific questions (lines 78-84):

Our model analysis addresses how the individual assessment of environmental actions combines with social interactions to determine the consistent adoption of proenvironmental behavior, and how this feeds back to the perceived environmental state. In particular, we ask whether larger costs of, or weak social pressure on, proenvironmental behavior make the consistent adoption of proenvironmental behavior less likely; and whether a slower pace of change in the perceived environmental state can promote consistency.

- The Results - Model Overview establishes the mathematical condition for the consistent adoption of a behavior (lines 153-168) (also see our response to your comment #4):

Even though the rates of behavior switching are deterministic functions, the individual switching events occur probabilistically. As a consequence, given that the population size is finite, the actual frequency of the behaviors fluctuates randomly, even asymptotically around the equilibrium values predicted by the deterministic model. In the Methods and Supplementary information, we show that the variance of the random fluctuations in frequency is equal to the total rate of behavior switching between A and B, elicited by social interactions and individual assessment of the environment. (...) We characterize A as being consistently adopted when the A equilibrium frequency, x^*, is high (close to one) and the corresponding total rate of behavior switching, $\Omega(x^*, e^*)$, is low (close to zero).

- The Discussion is now mapped with subheadings to highlight the main findings:The importance of timescales for behavioral consistency (line 305); Consistent adoption of incrementally more active behavior (lines 340); Limits and perspectives (lines 373). The Discussion also ends with a new paragraph of Concluding remarks, where we distill the main results and highlight important empirical questions raised by the results (lines 422-435):

In this minimal model of behavior-environment feedback, individuals intend proenvironmental action when they perceive their environment as degraded, and relax their effort as they perceive environmental improvement. This negative environmental feedback, by itself, opposes the consistent adoption of costly proenvironmental behavior, but opens the possibility for such behavior to rise from very low frequency and reach a tipping point at which social interactions and conformism will pick up and drive its consistent adoption. The theoretical conditions for this to occur (social interactions timescale faster than individual assessment timescale, strong social pressure for proenvironmental action) raise empirical questions regarding why individuals' environmental intentions would rely more on others' influence than on their own assessment of the environmental state, and what factors (individual or collective) can make the social pressure of conformism stronger for proenvironmentalism.

2. I found only 5 references to other human-environment models the authors use to discuss and compare their work against. A more thorough comparison with the literature on human-environment modeling is needed. Especially, works on evolutionary game theory and individual learning dynamics with environmental feedback are closely related to the authors' modeling approach. Embedding their model in this literature would help to better contextualize and relate it.

We agree that relating our model to the framework of evolutionary games with environmental feedbacks (or eco-evolutionary games) is quite relevant and interesting. The reason why we did not do this in the original manuscript (beyond referring to specific studies where the modeled behavior explicitly pertains to proenvironmentalism) relates to one key feature of our model – the treatment of individual assessment of the perceived environment and social interactions as two separate factors of individual decisions. In evolutionary games with environmental feedbacks, the environment influences the payoffs of the strategic interactions. In contrast, our model assumes that the payoffs are constant and that the perception of the environmental state can influence an individual's behavioral choice independently of their interactions with others. This structure allows us to evaluate the behavioral effect of individual assessment of the environment independently of social interactions, thus fitting the conceptual framework of Schill et al. (2019) (reference 42).

We added five general references to introduce evolutionary games with environmental feedbacks and discuss how our model relates to them: McNamara 2013 (ref. 34), Weitz et al. 2016 (ref. 50), Gokhale & Hauert 2016 (ref. 17), Hauert, Saade & McAvoy 2019 (ref. 22),

and Tilman, Plotkin & Akçay 2020 (ref. 47). The framework of evolutionary games with environmental feedbacks is introduced early on (lines 55-59):

Mathematical modeling of human-environment dynamics can help identify pathways toward proenvironmental behavior consistency. Game-theoretic models with environmental feedbacks, or eco-evolutionary games, have been developed to study coupled environmental and behavior dynamics [34,17, 22, 47, 50]. Specific examples address the use of a natural resource such as farmland [14], water [45] or forest [5].

We then explain how our model differs (lines 59-69):

A key aspect of these behavior-environment models [9, 48, 50] based on 'imitation dynamics' [24] is that individuals' behavioral decisions are only made in the context of their interaction with others. Yet factors that are not tied to social encounters, such as the individual's own experience and perception of the environmental state, may play a key role in environmental decisions. This is core to the conceptual framework that Schill et al. (2019) [42] recently put forth, building on behavioral economics and cognitive psychology, for understanding human behavior in the face of sustainability challenges. In this framework, individuals create social and environmental contexts that change dynamically with continuous feedback to their behavior. Following Schill et al. (2019) [42] we hypothesize that the dynamics of environmental behavior are shaped both by social context and the individuals' private environmental experience.

This important point is repeated in the opening paragraph of the Discussion (lines 294-303):

The treatment of individual assessment of the perceived environment and social interactions as two separate factors of individual decisions differentiate our model from evolutionary games with environmental feedbacks, in which the influence of the environment is entirely mediated by the payoffs of the strategic interactions [34,17, 22, 47, 50]. In contrast, our model assumes that the payoffs are constant and that the perception of the environmental state can influence an individual's behavioral choice independently of their interactions with others. This structure allows us to separate the behavioral effect of individual assessment of the environment from the effect of social interactions. Our model thus aligns with the conceptual framework of Schill et al. (2019) [42] for connecting environmental behavior with both social and perceived biophysical contexts.

Another major difference is, as you point out in your Comment #3, that in our model, the environmental state variable represents perceived information, rather than a true measure of a physical component of the environment. As a consequence, the environmental state does not have its own intrinsic dynamics; it is entirely shaped by behavioral intentions or actions, in the same vein as Weitz et al.'s (2016) model (reference 50). This is now explained in lines 414-420:

Another significant difference with most previous human-environment models is that here the environment, being purely informational, does not have its own intrinsic dynamics. A similar

assumption was made by Weitz et al. (2016) [50] in a game-theoretic model where the environmental feedback was positive and governed by a tipping point.

In spite of the structural difference between eco-evolutionary games and our model, comparing the results is interesting. A major finding of eco-evolutionary game theory is that the dynamics of eco-evolutionary games is sensitive to the timescales involved (intrinsic environmental dynamics, environmental reactivity to human action, relative to the timescale of encounters), and to the incentives for switching behavior depending on the state of the environment. We reach parallel conclusions. In our model, rather than the incentives (payoffs) changing with the state of the environment, behavioral switches are possible in direct response to environmental change. Because of the decoupling between individual assessment and social interactions, we introduce an additional timescale for the individual perception of environmental change, which measures the strength of the environmental feedback on individual decision. This timescale plays a critical role in the model dynamics, highlighted in the Discussion subsection The importance of timescales for behavioral consistency, lines 314-325 (text similar to the original version, slightly shortened).

In comparison, the effect of the timescale of environmental reactivity is quantitatively more subtle, albeit similar to what is found in eco-evolutionary games – slow environmental reactivity creates a time lag between the environmental state being assessed by individuals, and the frequency of behaviors which influence behavioral switches via social interactions; behavior-environment cycles may occur as a consequence. The corresponding explanations are in lines 326-338 (text similar to the original version) in the Discussion subsection The importance of timescales for behavioral consistency.

3. Some model design choices remain opaque to me. For example, why does the environmental state have no influence on the payoff difference? The authors make the case that in their model, the environmental state only presents perceived information rather than the true state of a physical environment. But if payoff feedback from the environment is excluded from the model, what does that mean for the validity of the timescales considered? It is not plausible to me to assume that this feedback plays only a negligible role for medium or long time scales. I believe, this design choice deserves more attention during the derivation of the model.

As we recalled in our response to your Comment #2, a key feature of the model is to separate individual assessment from social interactions. Specifically, the environment feeds back to behavior via individual assessment, while behavior may also - and independently - change as a result of social interactions. The distinction between social feedback and (direct) environmental feedback is aligned with the framework advanced by Schill et al. (2019). Our model allows us to contrast the negative feedback of environmental assessment and positive feedback of social conformism and resolve their effects when they operate in concert.

As you point out, the primary justification of our assumption that the payoff difference remains constant (unaffected by variation in the environmental state variable) lies in the short timescale involved in the behavior-environment dynamics. The environmental state variable captures perceived information, shaped by behavioral intentions or actions. A natural assumption then is that the timescale of environment-behavior change is short relative to the timescale over which the true state of the physical environment changes. Under this timescale separation, the payoff difference remains constant as the perceived environmental information changes, without precluding change in payoffs over the slower timescale of a changing physical environment. This explanation is now provided in the Discussion, in the first paragraph (lines 303-316) of subsection The importance of timescales for behavioral consistency.

Finally, we note that change in the payoff difference may also occur as a response to the system reaching a new behavior-environment stationary state. This is actually implicit in our scenario for the consistent adoption of incrementally more active behavior, described in the last subsection of the Results (lines 258-291) and illustrated with Fig. 4. This scenario unfolds over a slow timescale set by convergence to the stationary state followed by variation in the active behavior. A response of the payoff difference to a change in the environmental stationary state may be mediated by institutional intervention. This is now explained in lines 407-414:

This representation of the environment allowed us to make the assumption that the payoff difference is insensitive to environmental change. Note, however, that a feedback of the environment to the payoff difference can be seen as implicit in our scenario for the consistent adoption of incrementally more active behavior. This could be due to institutional intervention responding - on a slow timescale - to change in the environmental stationary state, as defined here, and altering the payoff difference through incentives (reducing the payoff difference) or by promoting more effective behaviors that would also be more costly (increasing the payoff difference).

4. I appreciate the fluctuation analysis the authors perform from a mathematical point of view. But what can we learn from it?

Motivated by your comment and Comment #2 of Reviewer 3, we revisited the presentation of the fluctuation analysis. In fact, our macroscopic model really is the coupled set of ordinary differential equations that approximates the deterministic dynamics of behavior frequency and a Ornstein-Uhlenbeck stochastic process that describes the random fluctuations. Both deterministic and stochastic components are key to the mathematical characterization of behavioral consistency. This is now stated in the Abstract (lines 10-13):

We show that the deterministic dynamics and the stochastic fluctuations of the system can be approximated by ordinary differential equations and a Ornstein-Uhlenbeck type process. By

definition, the proenvironmental behavior is adopted consistently when, at population stationary state, its frequency is high and random fluctuations in frequency are small.

In the Results - Model Overview we now have a subsection entitled Deterministic approximation of the dynamics and stochastic fluctuations (starting at lines 136-137) where we explain the origin of stochastic fluctuations and state the key result (established in the Methods and Supplementary material) whereby the variance of the fluctuations in the behavior frequency is equal to the total rate of behavior switching across the population. See lines 153-168 (quote given above, in our response to your Comment #1).

This mathematical characterization of behavioral consistency led us to rephrase and clarify the conditions under which consistent adoption of the proenvironmental behavior is expected (subsection Spread and consistent adoption of costly active behavior) with an updated Figure 3 to show how the total rate of behavior switching (measuring the amount of random fluctuations around the equilibrium frequency):

The consistent adoption of a costly active behavior requires the equilibrium frequency, x^*, to be close to one and the total switching rate, Ω, to be close to zero. Figures 2 and 3 illustrate conditions under which this is the case. According to the previous subsection, a general condition for a costly (or low-incentivized) active behavior to spread from low to high frequency (x^* close to 1) is that the social pressure for A be strong enough (large δ_A hence small ν, Fig. 2). When that is the case, the product $\tau(l_B - l_A)$ fully determines consistency, since for x^* close to 1, the total rate of behavior switching is approximated by

$$\Omega = \tau(l_B - l_A).$$

Thus, with a relatively weak sensitivity to the environment (i.e. τ low) and a small environmental impact differential (i.e. l_A close to l_B), costly active behaviors can spread from low to high frequency provided the social pressure for active behavior is strong enough (Fig. 2a-c), and consistent adoption is expected (Fig. 3a).

When discussing the scenario of the consistent adoption of incrementally more active behavior, we also added a paragraph to point out the role that fluctuations may play in the case of coexisting alternate equilibria: if the system starts with a very low frequency of the active behavior and settles at a low-x^* equilibrium, fluctuations may take the population to visit the high-x^* equilibrium, transiently but long enough for a new, more active behavior to be adopted. This effect of fluctuations on the system dynamics in the bistable case is now illustrated with the new Figure 5:

We hypothesize that such an incremental scenario might be triggered even for active behaviors that are initially costly, and with weak social pressure for them (small δ_A hence high ν) -- as long as their cost is not too large so that bistability remains possible (upper areas of black regions in Fig. 4b). In this case, random fluctuations in behavior frequency may cause the system to switch from the low-frequency to the high-frequency A equilibrium (Fig.

5), where it may reside long enough for some new behavior A' more active than A to spread and take over.

5. The authors states "Environmental feedback tends to generate behavioral inconsistency, which eventually limits the environmental impact reduction of active behavior". I wonder whether the second part of this sentence is true. I can see that including environmental feedback in the behavioral adaptation processes in addition to the social norm component causes more mixed strategy equilibria (Fig 2, comparing tau=0.1 with tau=1.0). This means that previously fully active parameter regions are less active now. But this also means that previously full non-active parameter regions have become somewhat active. There, it may be better to be inconsistent with your pro-environmental behavior than to show no pro-environmental behavior at all.

This is a very good point. We modified the text to account for it as we describe the combined effect of social and environmental feedbacks on active behavior frequency. The corresponding text now reads (lines 213-223):

On the other hand, for large values of the product $\tau(l_B - l_A)$, the environmental feedback dominates the effect of social conformism for the active behavior. As a consequence, most active behaviors can reach a frequency close to 0.5 irrespective of their cost and the intensity of social pressure (Fig. 2g-i). High frequency (close to one) can be reached but only under very strong social pressure, even for active behavior that are strongly beneficial (large $\beta > 0$).

The important point then is that the internal "mixed strategy" equilibrium is associated with large fluctuations, as shown in Fig. 4a and g. Thus, the reduction in environmental impact measured by the equilibrium e* in Fig. 4d is deceptive, in the sense that the actual environmental impact is expected to fluctuate widely, as the behavior frequencies themselves fluctuate around their equilibrium. The point is made in the subsection Spread and consistent adoption of costly active behavior (lines 245-250):

In contrast, if the individual sensitivity to the environment is strong enough (large τ) and/or the environmental impact differential is large (large $l_B - l_A$), behavioral inconsistency is expected, with agents frequently switching between active and baseline behaviors (Fig. 3b-c), even if the predicted equilibrium frequency of the active behavior is high (see lower (yellow) regions in Fig. 2d-i, where the social pressure for A, δ_A, is high hence the social normal threshold, ν, is low).

6. The authors' model interpretation regarding the question of behavioral consistency is as follows: "If x converges to 1, individuals perform behavior A most of the time, which means that the individual switching rate from A to B vanishes. In other words, behavior A is expressed consistently. If x converges to 0, the individual switching rate from B to A vanishes and behavior B is expressed consistently. In all other cases, individual behavior is

inconsistent." Let me challenge this interpretation. Imagine a standard (one-population) imitation/replicator dynamics in the chicken/hawk-dove/snowdrift game. The dynamics converge to a mixed strategy, but does this represent behavioral inconsistency? Isn't it likewise plausible to assume that a fraction of the population consistently plays one strategy and the rest of the population plays the other?

We agree that the macroscopic deterministic model, equation (1), cannot make the difference between one single plastic phenotype (i.e. all individuals alternating between two behaviors) and a mixture of two constant phenotypes (i.e. one constantly expressing one behavior and the other constantly expressing the other behavior). However, our microscopic model assumes the former and excludes the latter: there is only one type of agents, they all perceive their environmental and social contexts in the same way, and in response can switch between behaviors A and B. Thus the frequency equilibrium and fluctuations of the macroscopic model measure the population-level outcome of individuals who keep switching between behaviors, often if the fluctuation variance is large, rarely if the fluctuation variance is small. This is now clarified as we explain how the fluctuation analysis is used to characterize behavioral consistency (lines 153-164, see also our response to your Comment #1).

Minor comments

* The references to the figures from the main text can be improved. For example, the first time the reader sees a reference to Figure 1 is when they are encouraged to compare Figure 1 with Figure 2.

Thank you for pointing this out. We carefully checked how the figures are called in the text. For example, we revised the caption of Figure 1 to better link it with the result of section “Behavior dynamics without environmental feedback” (starting at line 175).

* When introducing a new model it can only help to have descriptive parameter names, as the authors have. The authors could improve their presentation of the figures and the text by naming the descriptive parameter names in addition to the corresponding symbol.

We heeded your suggestion to make sure that for each parameter, the text includes both the parameter name and corresponding symbol.

Reviewer #2

This paper addresses some current issues relevant to everyone's life. By considering a coupled system for the environment and the social public goods game, the authors explore the model through theoretical and numerical analysis, trying to reach meaningful conclusions to guide policymaking and the general public. It is good for publication in my opinion. Here are some questions and comments on minor issues.

We thank you for your interest and support and careful read of the manuscript. We made all the changes that you suggested.

Page 2 Abstract line 11: "little sensitive", maybe change to "indifferent"

Done.

Abstract line 15: "Noticingly", should be "Noticeably"

Done.

Page 3 Introduction line 5: remove "to make"

Done.

Page 4 line 2: "change dynamically with and feed back continuously", change to "change dynamically with continuous feedback"

Done.

Page 4 Results Model overview line 2: the variable E and later the symbol e seem to both represent the environmental state. Maybe the authors should try to pick one of them and be consistent with the notations.

We corrected this. We note, however, that the notation E (and X) is relevant in the description of the stochastic model, while notations e and x for the environment and behavior state variables are introduced in the deterministic approximation – see the subsection Deterministic system approximation for large populations starting at line 506 in the Methods.

Page 4 Results Model overview line 3: "with larger E meaning that the environment is perceived as more degraded", change to "with larger E as more degraded environment"

Done.

Page 5 lines 2 and 3: the A behavior, the B behavior, can be replaced by behavior A and behavior B

Done.

Page 6 line 2 from the bottom up: "was allowed to arise" change to "arose"

We rephrased the sentence.

Page 7 line 5: I believe the fixed point x^* should be (\delta_B-\beta)/(\delta_A+\delta_B).

Thank you for noticing it. We corrected the typo.

Page 7 Effect of environmental feedback on behavior dynamics Equation (4): What is p(x) here? What is p_0(x) here? Are they supposed to be the right-hand side of the first equation in equation (2)? Maybe add that e is always positive, so the only option for equilibrium is e=l_A x+l_B(1-x)

We added the clarifications needed around equation (1), lines 141-152, and equation (5), line 179.

Page 8 line 2: Figure 1 and 2. I'm not sure what the format requirement of the journal is, but it would be very helpful if the figures mentioned are actually presented on the same page here

Thank you for pointing this out. Should the manuscript be accepted for publication, we shall see at formatting stage if this is possible.

Page 8 Conditions for spread and consistency of active behavior line 3: "can be propagated and stabilized" change to "can propagate and stabilize"

We rephrased the sentence as you suggested.

Page 8 Conditions for spread and consistency of active behavior line 6: "and adopted" change to "and be adopted"

Done.

Page 9 line 8: rewrite "drive the rise of A frequency close to 1"

The sentence was rephrased.

Page 9 line 14: add "l" after "environmental reactivity"

Done.

Page 9 line 2 and 4 from the bottom and the section title: "stationary" misspelled

Done.

Page 10 line 3: "behavioral" change to "behavior"

Done.

Page 10 paragraph 3 line 3: E^* has not been defined anywhere

Thank you for pointing this out. Equilibrium points are now defined at lines 151-152.

Page 11 line 2: typo "further"

Corrected.

Page 14 bottom two lines: rewrite "We refer to ...Table 1"

Done.

Page 15 Social interactions equation (6): why is the right hand side only dependent on j? shouldn't it depend on both i and j just like in equation (10)? Also what would be the formula for i to i and j to j?

We added a sentence (lines 462-463) after former equation (6), now equation (9), to explain why only a dependence on j appears. In fact, i and j symmetrically contribute to the number of potential encounters accounted for in equation (9). The interaction rate for i-to-i (and similarly j-to-j) would not contribute to the dynamics because the frequency does not change from this interaction. This can be seen in equation (14), where we would have (f(x,e)-f(x,e)) as multiplier for an interaction term corresponding to i-to-i encounters.

Page 16 lines under equation (10): rewrite "tau_A and tau_B must capture ... , compared to the alternative behavior". maybe try to explain what tau_A and tau_B represent first, and then describe what rule/law they should follow

Thank you for this suggestion. We rewrote this part, see lines 472-478. We now first explain what $\tau_A(E^N)$ and $\tau_B(E^N)$ describe, next we introduce equation (13), and finally we give the interpretation – why the switch depends on the gap between E^N and l_A or l_B.

Page 17 lines 2 and 3: Maybe add a little explanation to what f(x,e) represents.

We added an explanation (lines 500-502) about how specifying f(x,e) in equation (14) yields the descriptors of the dynamics that we are looking for (deterministic part and stochastic fluctuations), as can be seen in supplementary equations (2) and (4).

Page 17 line above Quantifying the effect of individual stochasticity: Plug equations (14), (15) and (1) into (13), we get equation (2).

Done.

In the supplementary material, section 3.1, equation (8) has some minor errors, I think maybe the rescale r should just be l over tau, there is no l_B, and let c just be l_A/(l_B-l_A), then the right hand side of the second equation of (8) would be r(v+c)(1-x-v). Because of this, the Jacobian in section 3.2 also needs some modification.

We thank the reviewer for detecting this typo. The rescaled r was l over τ times c and not l over τ times l_B. This is now corrected (line 60 in the Supplementary material) and we detail

the calculation (lines 66-67). As it was only a typo, this does not affect our analysis and the results are unchanged. We also thank the reviewer for suggesting an alternative rescaling. We decided to keep our original choice, which was effective enough to conduct the analysis.

Supplementary material Section 3.4 line 2: equation (18), I don't think there is an equation (18) in the supplementary material

This was corrected.

Reviewer #3

This paper presents an extension of the family of "bioeconomic" models for natural resource management developed in the 1970's by Colin Clark (see Mathematical Bioeconics, 1976, 1990, 2005). The extension adds 'social interaction'(replicator dynamics) between two behavior types that determine environmental impact (harvest in bioeconomic models). The paper reports on the impact of various assumptions about model parameters on the characteristics of equilibria.

We thank you for your comments. We revised the language throughout in response to your Comment #1, clarified our definition of behavioral consistency (your Comment #2), and streamlined the Discussion, including a stronger conclusion (your Comment #3).

1. As such, I struggle a bit with stretching such a simple model to describe 'Behavior dynamics' and 'social interactions'. Yes, the model describes the behavior of very, very simple algorithms, not humans. I think the paper should soften language. i.e. this line on page 15 (there are no line numbers so the page number is the best I can do.....)

"Two factors influence individual behavior: social interactions and individual assessment of the environmental state."

This sounds like we are talking about sentient beings. This is misleading. Something like

"Two factors influence the decision process of modeled agents: frequency of encounters with other modeled agents, and the value of the state variable representing some environmental factor."

is more accurate and gives a sense to the reader what the paper is actually about. It is not really about human-environment feedback and the consistency of proenvironmental behavior, but rather, a very simple model of interacting particles that captures some general features of human-environment feedback and the consistency of proenvironmental behavior. I think the title should include the world modeling, simulating, etc. to more accurately describe what the paper is about.

We heeded your suggestion. We used the language that you suggested for that particular sentence (lines 109-110 and 120-121) and strived to carry out the sense of highly simplified representation of behavior throughout our manuscript. For example, we used "individuals" and "social interactions" in our description of the biological underpinning of the model, and "agents" and "encounters" in our description and analysis of the mathematical model and simulations. We tried to balance the systematic use of such language with the legibility and conciseness of the writing.

2. Along the same lines, the links between the language used to describe model behavior and the model itself is sometimes not clear. I searched 'behavioral consistency' in the PDF document. To the extend I can rely on the search results, it appeared 11 times, yet it is never explicitly defined. Of course, I can guess what it is, but it would be nice to be able to confirm my understanding with that of the authors.

We thoroughly revised our presentation and analysis of behavior consistency in response to your comment and Reviewer 1's Comments #1 and #4. Hereafter we recall some elements of our response to Reviewer 1.

Behavior consistency is now explicitly defined in the Abstract (lines 10-13):

We show that the deterministic dynamics and the stochastic fluctuations of the system can be approximated by ordinary differential equations and a Ornstein-Uhlenbeck type process. By definition, the proenvironmental behavior is adopted consistently when, at population stationary state, its frequency is high and random fluctuations in frequency are small.

A verbal description of behavior inconsistency is given early in the Introduction (lines 40-42):

Rather, non-ideologically polarized individuals tend to show inconsistent behavior as they change opinion, revise their intention, or switch behavior during their lifetime [1], possibly on very short timescales [8].

The formal characterization of behavior consistency is introduced at the end of the Introduction (lines 76-78):

We use the model to investigate the determinants of proenvironmental behavior consistency. We say that a behavior is adopted consistently when its frequency at stationary state is high and the stochastic fluctuations in frequency are small.

The mathematical characterization is obtained in the Results - Model overview (lines 153-168):

Even though the rates of behavior switching are deterministic functions, the individual switching events occur probabilistically. As a consequence, given that the population size is finite, the actual frequency of the behaviors fluctuates randomly, even asymptotically around the equilibrium values predicted by the deterministic model. In the Methods and Supplementary information, we show that the variance of the random fluctuations in frequency is equal to the total rate of behavior switching between A and B, elicited by social interactions and individual assessment of the environment. (...) We characterize A as being consistently adopted when the A equilibrium frequency, x^*, is high (close to one) and the corresponding total rate of behavior switching, $\Omega(x^*, e^*)$, is low (close to zero).

Results are now rooted in this mathematical characterization, see for example Results - Spread and consistent adoption of costly active behavior. The Figure 3 was revised to provide more information about how the total rate of behavior switching varies with key model parameters, and the new Figure 5 was added to illustrate the effect of individual behavior switching on the system dynamics in the case of coexisting alternate equilibria (bistability). The significance of this effect is discussed in a new paragraph at the end of the subsection Results - Robust environmental impact reduction through incremental behavior change (lines 284-291).

3. Finally, the discussion is a bit long and technical with many suggestions for further research. Perhaps readers of this journal will appreciate this. For my part, given the simplicity of the model and rather intuitive nature of the results, I am missing a good punch line or some sort of interesting empirical puzzle that this analysis sheds new light on. Yes, this is the journal of computational biology, but some more interesting motivation for and insights from the computation would improve the paper substantially.

We are grateful that you gave us the impetus to revise the Discussion substantially, to make it shorter, with a clearer structure (marked with subheadings). The suggestions for future research are now gathered in one subsection, Limits and perspectives (lines 373-420). We completely rewrote the conclusion, to emphasize the main theoretical finding and to outline a couple of interesting empirical questions raised by the theory (lines 423-435):

In this minimal model of behavior-environment feedback, individuals intend proenvironmental action when they perceive their environment as degraded, and relax their effort as they perceive environmental improvement. This negative environmental feedback, by itself, opposes the consistent adoption of costly proenvironmental behavior, but opens the possibility for such behavior to rise from very low frequency and reach a tipping point at which social interactions and conformism will pick up and drive its consistent adoption. This suggests a scenario to achieve large reductions in environmental impact, which involves the consistent adoption of incrementally more active and potentially more costly behavior. The theoretical conditions for this to occur (social interactions timescale faster than individual assessment timescale, strong social pressure for proenvironmental action) raise empirical questions regarding why individuals' environmental intentions would rely more on others' influence than on their own assessment of the environmental state, and what factors (individual or collective) can make the social pressure of conformism stronger for proenvironmentalism.
