# Supplementary material for: Human-environment feedback and the consistency of proenvironmental behavior
Source: PLoS Comput Biol. 2023 Sep 18;19(9):e1011429. doi: 10.1371/journal.pcbi.1011429 (PMC10538744; doi:10.1371/journal.pcbi.1011429)
Supplement: Attachment — Submitted filename: Retour des rapporteurs Article PLOS -2.pdf [file pcbi.1011429.s005.pdf]

Dr. Christian Hilbe
Academic Editor, *PLOS Computational Biolog*y

Dr. James O'Dwyer
Section Editor, *PLOS Computational Biology*

Ref.: Manuscript PCOMPBIOL-D-22-01352

July 17, 2023

Dear Dr. Hilbe and Dr. O'Dwyer,

We thank you for gathering a second round of comments from the Reviewers of our manuscript "Human-environment feedback and the consistency of proenvironmental behavior" and for giving us the opportunity to submit a second revision.

Reviewer 3 was satisfied with our response and revision. Reviewer 2 only asked for one sentence to be rephrased, that we did. Reviewer 1 raised three additional points. By carefully addressing them, we believe that our manuscript further gained in clarity. Their first point focuses on how we model the environmental state as perceived by individuals. We clarified our definition of the environmental state variable as driven by the population environmental impact. The second point is about timescales – what is the relevant timescale of our model, given our assumption of a constant environment? This point prompted us to further clarify what we mean by 'constant environment' in order to dissipate any ambiguity. In the case of global warming, for example, the physical environment is assumed to be 'constant' in the sense that the warming trend is unaffected by the individual behaviors over the model timescale – in line with the fact that our world is locked in a warming trend which would remain unabated for decades even if we were to stop all our greenhouse gas emissions today. Taking the decadal timeline as reference for the characteristic convergence time of our model, we gathered exemplary simulations to show that one week or one month can be used as unit times that are consistent with that timeline and the behavioral processes that the model describes. Third, the Reviewer suggested that we include additional references to models of behavioral choice that involve experiential learning. The Reviewer kindly provided us with a list of such references, that we carefully reviewed and commented on in our point-by-point response. In our revision, we cited those references that were most directly related to the motivation and scope of our work.

Hereafter we include our detailed point-by-point response to the Reviewers' comments. In our revised manuscript all changes appear in red. We updated the figures and the supplementary material (including the new supplementary figure 5). Also, we checked the accessibility of the code and readme file that we uploaded on the PLOS platform.

We hope that our response and revised manuscript meet your expectations and we look forward to your decision.

Sincerely,

Claire Ecotière (corresponding author)
Jean-Baptiste André
Régis Ferrière
Sylvain Billiard
Pierre Collet
Sylvie Méléard

****

Point-by-point response

We greatly appreciate your consideration of our responses and revisions, and additional comments. Hereafter your comments appear in black; our responses are in blue, and quotes from the revised manuscript are in purple. We thank you again for the time you devoted to evaluate our work and for your thorough and constructive comments.

Reviewer #1

The authors addressed all of my comments in the revised version of the manuscript. Now it becomes much more apparent that the model assumes the behavior is driven directly by environmental perception and not a payoff difference resulting from environmental changes.

Three remaining pointers concern the model's interpretation and validity.
- In the model, the perception of the environmental state is proportional to the pro-environmental active individuals. Why should the individual's private environmental experience depend on the behavior of others when considering the actual state of the environment as not changing? I can only think of societal movements or cultural trends, like climate protests, plant-based diets, or flight shaming, where such a dynamic seems plausible. But these are purely social processes without environmental feedback - precisely because the environment is not changing.

We essentially agree. Your question prompted us to revisit how we define the environmental variable *E* (or *e*) and how we explain its dependence on the behaviors frequencies and individual impacts (measured by parameters *I_A* and *I_B*). *E* (or *e*) is intended to quantify an overall level of environmental degradation or vulnerability as perceived by each individual. As you suggest, this perceived environmental degradation or vulnerability may change as a function of, for example, the intensity (frequency, distribution, size) of climate protests or of the denunciation (such as flight shaming) of environmentally detrimental behavior, or the popularity of plant-based diets, the use of public transportation, the practice of reusing and recycling, or the reduced consumption of non-essential goods or services. Thus, the state variable *E* (or *e*) variation is driven by the frequencies of the different behaviors and the perceived strength of the intention or action. The state variable *E* may then be seen as a summary statistics or indicator, experienced or communicated via some information channel that makes it available to all individuals in the modeled population. Accordingly, we made the following changes in our revision. In Results - Model overview, we added (lines 110-114):

The *e* variable can be seen as an indicator or summary statistics of the perceived level of environmental degradation, whose variation is driven by the population level of environmental action, intention, or awareness, such as the spread of renewable energy, the adoption of plant-based diets, the reduced consumption of non-essential goods, or the prominence of pro-environmental demonstrations and other public calls for proenvironmental action.

Throughout the manuscript, we favored using the phrase perceived environmental degradation to clarify the meaning of the environmental state variables *E* and *e*.

- The choice of modeling constant environments has consequences for the validity of the model's time range. Letting the social process run for longer than one assumes the environment to be constant makes the model inconsistent. For example, in the face of global change, we cannot consider the environment constant on time scales longer than decades. This would mean that all social processes in the model must operate on time scales much shorter than decades - let's say on a yearly time scale. What time interval does a model step represent? A day? How long does it take until equilibrium is reached? It would help greatly if the authors estimate the actual timescales (days, weeks, months, years, decades) on which their model operates.

Thank you for prompting us to further clarify the model timescale. We felt that another important clarification, also relevant to your previous point, was needed around what we mean by a “constant environment”. Considering climate warming as a central example, we are not looking at a time scale over which warming is negligible. Rather, we are assuming a timescale over which the trend of global environmental degradation (e.g. warming) is essentially unaltered by individual behaviors. Our approach assumes that our world is already locked into warming: even if we stopped emitting GHG today, it would take several decades before we see curbing in the rise of global temperature. In contrast, we may see rapid change in individual behavior, including the spread of proenvironmental behavior, concomitantly with an increase in the overall effect of proenvironmental action or intention, such as a decrease in non-essential goods consumption, or even in GHG emissions (see our response to your previous point for examples).

In our model, the timescale for such changes is set by parameters kappa, tau, and *l*. In our revision, we added typical simulations to illustrate how long it takes for the frequency of proenvironmental behavior to rise from near zero to near one in a well-mixed population. This is the new section 3.5 in the supplementary material and new supplementary figure 5. With kappa = 1 (i.e. one social interaction about the environmental concern expected on average per unit time), this is typically of the order of 1-50 unit time. Thus, environment-related social encounters that happen on average once a week or once a month would be consistent with the typical dynamics of the model. With a one-week time unit, the individual assessment of the environment would occur on average every three months with tau = 0.1, or roughly every day with tau = 10. With a one-month time unit, individual assessment of the environment would occur, on average, roughly every year with tau = 0.1, and every three days with tau = 10. Over such timescales, convergence to the stationary state occurs well before the actual environmental trend (e.g. speed of warming) might change as a consequence of the population consistently adopting proenvironmental behaviors.

Accordingly, we made the following changes in the manuscript. In the Results - Effect of environmental feedback on active behavior frequency, we added the following paragraph (lines 255-270):

Simulations of trajectories in the case of the active behavior rising from low to high frequency allow us to constrain the model unit time (supplementary figure 5). Our approach assumes a timeline over which the global environmental state or trend of global environmental degradation (e.g. climate warming) is essentially unaltered by individual behaviors. The

timescale over which individual behavior changes is set by parameters κ, τ, and I. With $\kappa = 1$ (i.e. one social interaction about the environmental concern expected on average per unit time), the characteristic time for the frequency of proenvironmental behavior to rise from near zero to near one is of the order of 1-50 unit time (supplementary figure 5). Thus, environment-related social encounters that happen on average once a week or once a month would be consistent with the typical dynamics of the model. With a one-week time unit, the individual assessment of the environment would occur on average every three months with $\tau = 0.1$, or roughly every day with $\tau = 10$. With a one-month time unit, individual assessment of the environment would occur, on average, roughly every year with $\tau = 0.1$, and every three days with $\tau = 10$. Over such timescales, convergence to the stationary state occurs well before the actual physical environment or environmental trend (e.g. speed of warming) might change as a consequence of the population consistently adopting proenvironmental behaviors.

In the Discussion - Limits and perspectives, after the sentence “we assume that the [physical environmental] change would occur on a much slower timescale and therefore has no influence on the individual decisions that the model describes.”, we added (lines 444-449)

For example, in the case of global climate change, our approach assumes that our world is already locked into warming: even if we stopped emitting greenhouse gases today, it would take several decades before we observe curbing in the rise of global temperature. None-the-less, rapid change in individual behavior may occur, including the spread of pro-climate action or intention, concommitently with the perception that the population environmental impact improves.

We also updated Table 1 to include units in the parameters description.

- The authors distinguish their model from evolutionary games with environmental feedback in the feature of perceived environmental and social interactions as two separate factors of individual decisions - referencing Schill et al., who argue for connecting environmental behavior with both social and biophysical contexts. However, behavioral choice independent of interactions with others has also been operationalized with models of individual learning. See, for example, single-agent models:
- Lindkvist, E., & Norberg, J. (2014). Modeling experiential learning: The challenges posed by threshold dynamics for sustainable renewable resource management. _Ecological Economics_, _104_, 107-118.
- Lindkvist, E., Ekeberg, Ö., & Norberg, J. (2017). Strategies for sustainable management of renewable resources during environmental change. _Proceedings of the Royal Society B: Biological Sciences_, _284_(1850), 20162762.
- Strnad, F. M., Barfuss, W., Donges, J. F., & Heitzig, J. (2019). Deep reinforcement learning in World-Earth system models to discover sustainable management strategies. _Chaos: An Interdisciplinary Journal of Nonlinear Science_, _29_(12), 123122.
- Chapman, M., Scoville, C., Lapeyrolerie, M., & Boettiger, C. (2022). Power and accountability in reinforcement learning applications to environmental policy. _arXiv preprint arXiv:2205.10911_.
but also more recently in multi-agent situations:
- Barfuss, W., Donges, J. F., Vasconcelos, V. V., Kurths, J., & Levin, S. A. (2020). Caring for

the future can turn tragedy into comedy for long-term collective action under risk of collapse. _Proceedings of the National Academy of Sciences_, _117_(23), 12915-12922.
- Huang, F., Cao, M., & Wang, L. (2020). Learning enables adaptation in cooperation for multi-player stochastic games. _Journal of the Royal Society Interface_, _17_(172), 20200639.
- Zhang, T., Williams, A., Phade, S., Srinivasa, S., Zhang, Y., Gupta, P., ... & Zheng, S. (2022). AI for Global Climate Cooperation: Modeling Global Climate Negotiations, Agreements, and Long-Term Cooperation in RICE-N. _arXiv preprint arXiv:2208.07004_.

We very much appreciate you providing us with these insightful references. We added Lindkvist & Norberg (2014) to relate our assumption of individual environmental assessment to models of behavioral choice driven by experiential learning. Regarding multi-agent models that address individual learning, we added references to Huang et al. (2020) and Barfuss et al. (2020). We note, however, limitations in the way learning is included in Barfuss et al. (2020); and we note in both Huang et al. (2020) and Barfuss et al. (2020) essentially the same conceptual differences as with other game-theoretical models with environmental feedbacks.

Specifically, Huang et al. (2020) and Barfuss et al. (2020) develop stochastic game models of social dilemmas in which the actors' actions influence the state of the environment, 'prosperous' versus 'degraded', and the environmental state feeds back to the game's nature and payoff. The prosperous state is associated with a public goods game (PGG) in which defection is expected to dominate, while the degraded state is modeled as an equal loss imposed on all players (Barfuss et al. 2020) or by prescribing a game type with known outcome (Huang et al. 2020), e.g. a PGG with dominating defection, inverse PGG with dominating cooperation, stag-hunt with bistability, or snowdrift with coexistence.

In Barfuss et al. (2020), learning dynamics are considered essentially "as a microfoundation to explain the emergence of cooperation or defection", i.e. "a technical method to confirm and refine the equilibria analysis" whereby the replicator equation can be used to model the time dynamics of actors' behavior (the probability of playing cooperation or defection given the environmental state). The main result of their equilibrium analysis, that is confirmed by the analysis of learning dynamics, is that "under a sufficiently severe and time-distant collapse, how much the actors care for the future can transform the game from a tragedy of the commons into one of coordination, and even into a comedy of the commons in which cooperation dominates." They emphasize that in their model "actors are interpreted best as representatives of geopolitical units, such as states, unions of states, or cities". Altogether, individual learning is not explicitly modeled as a behavioral property of agents, and the ingredients (risk of evironmental collapse affecting the population game and individual payoffs, and future rewards discounting), make the nature and scope of their model rather different from ours.

Huang et al. (2020) implement actor-critic reinforcement learning explicitly at the level of individuals. The environmental state influences the game type and payoffs, but there are no specific rules to update the environmental state. Rather the model assumes that the environment follows a stationary probability distribution. This assumption is relaxed in an extension of the model, where the probability of the environment being in the prosperous vs. degraded state changes at a rate that is influenced by the frequency of cooperators. This

extension makes it possible to address the joint dynamics of cooperation and the environmental state, as we do in our study. The authors conclude, “although our model is stochastic and incorporates the effect of environment and learning, we can still observe those dominance, bistability and coexistence behaviours analogously obtained under the deterministic replicator dynamics” but provide few details into the outcomes, e.g. whether there exist conditions under which high levels of cooperation may be established in spite of the environmental feedback. The question of stochastic fluctuations around the stationary state is not addressed.

Just as in the case of ecoevolutionary games, our model is fundamentally different from stochastic games such as Huang et al. (2020) or Barfuss et al. (2020), as we assume that the economic favorability of cooperation (active behavior) vs. defection (baseline behavior) is unaffected by the perceived population environmental impact. We show that even when consistent defection is favored in the absence of environmental feedback (negative payoff differential), individuals processing information about the population environmental impact can promote enough B→A behavioral switches to drive the frequency of active behavior A above a social tipping point, leading to a stationary state with high A frequency and small stochastic fluctuations.

Accordingly, we made the following changes in our revision. In the opening paragraph of the Discussion, we cited Huang et al. (2020) and Barfuss et al. (2020) (line 335) and included the reference to Lindkvist & Norberg (2014) as follows (lines 335-339):

Here we assume that the payoffs are constant and that the perception of environmental degradation can influence an individual’s behavioral choice independently of their interactions with others – a similar assumption is made in models of environmental behavioral choice based on experiential learning [LINDVIKST AND NORBERG 2014].

Reviewer #2

The revision added explanations to parameters in the model and general conceptual understanding of the model. I think it is ready for publication after rewriting "Individuals perceive having an impact on the environment to a degree that is determined by their behavior" on page 4 lines 88-89.

Thank you for your positive assessment. We rephrased the sentence accordingly: “Each individual has a negative impact on their environment that depends on their behavior – the impact of the active, proenvironmental behavior being less than the impact of the baseline behavior.”

Have the authors made all data and (if applicable) computational code underlying the findings in their manuscript fully available?
The PLOS Data policy requires authors to make all data and code underlying the findings

described in their manuscript fully available without restriction, with rare exception (please refer to the Data Availability Statement in the manuscript PDF file). The data and code should be provided as part of the manuscript or its supporting information, or deposited to a public repository. For example, in addition to summary statistics, the data points behind means, medians and variance measures should be available. If there are restrictions on publicly sharing data or code —e.g. participant privacy or use of data from a third party—those must be specified.

Reviewer #1: **No:** The PDF said so in the beginning, but I could not find an actual Data or Code Availability Statement and, therefore, was not able to look at the code.

Reviewer #2: Yes

Reviewer #3: None
